# Application of environmental DNA metabarcoding to identify fish community characteristics in subtropical river systems

**DOI:** 10.1002/ece3.11214

**Published:** 2024-05-09

**Authors:** Sai Wang, Dong‐Hai Wu, Yong‐Duo Song, Tuan‐Tuan Wang, Shi‐Di Fan, En‐Ni Wu, Nan‐Lin Chen, Wen‐Tong Xia, Min N. Xu, Zhong‐Bing Chen, Jing Wen, Yang Zhang, Ling Mo, Lei Xiang

**Affiliations:** ^1^ State Key Laboratory of Marine Resource Utilization in South China Sea Hainan University Haikou China; ^2^ College of Ecology and Environment Hainan University Haikou China; ^3^ Key Laboratory of Agro‐Forestry Environmental Processes and Ecological Regulation of Hainan Province Hainan University Haikou China; ^4^ Department of Applied·Ecology, Faculty of Environmental Sciences Czech University of Life Sciences Prague Praha‐Suchdol Czech Republic; ^5^ Hainan Qingxiao Environmental Testing Co., Ltd. Sanya China; ^6^ Hainan Qianchao Ecological Technology Co., Ltd. Sanya China; ^7^ Shenzhen Guanghuiyuan Environment Water Co., Ltd. Shenzhen China; ^8^ Hainan Research Academy of Environmental Sciences Haikou China; ^9^ Department of Ecology Jinan University Guangzhou China

**Keywords:** biomarker, fish community, high‐throughput sequencing, metabarcoding, monitoring tool, river food web

## Abstract

Fish are vital in river ecosystems; however, traditional investigations of fish usually cause ecological damage. Extracting DNA from aquatic environments and identifying DNA sequences offer an alternative, noninvasive approach for detecting fish species. In this study, the effects of environmental DNA (eDNA), coupled with PCR and next‐generation sequencing, and electrofishing for identifying fish community composition and diversity were compared. In three subtropical rivers of southern China, fish specimens and eDNA in water were collected along the longitudinal (upstream–downstream) gradient of the rivers. Both fish population parameters, including species abundance and biomass, and eDNA OTU richness grouped 38 sampling sites into eight spatial zones with significant differences in local fish community composition. Compared with order‐/family‐level grouping, genus‐/species‐level grouping could more accurately reveal the differences between upstream zones I−III, midstream zones IV−V, and downstream zones VI–VIII. From the headwaters to the estuary, two environmental gradients significantly influenced the longitudinal distribution of the fish species, including the first gradient composed of habitat and physical water parameters and the second gradient composed of chemical water parameters. The high regression coefficient of alpha diversity between eDNA and electrofishing methods as well as the accurate identification of dominant, alien, and biomarker species in each spatial zone indicated that eDNA could characterize fish community attributes at a level similar to that of traditional approaches. Overall, our results demonstrated that eDNA metabarcoding can be used as an effective tool for revealing fish composition and diversity, which is important for using the eDNA technique in aquatic field monitoring.

## Graphical abstract

Environmental DNA (eDNA), coupled with PCR and next‐generation sequencing, and electrofishing were used to compare their effects in identifying fish community characteristics. Our results demonstrated that eDNA metabarcoding can be used as an effective tool in revealing fish composition and diversity, which is important for using the eDNA technique in aquatic field monitoring.

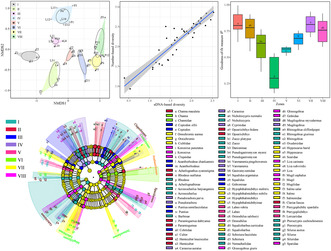

## 1 INTRODUCTION

Large river systems are essential for providing critical foraging, breeding grounds, and nursery habitats for a variety of fauna and are considered among the most productive ecosystems worldwide (Wang, Luo, et al., 2020; Wang, Wang, Xia, et al., 2021). Fish, as consumers at high trophic levels in river food webs, represent the sum of a wide range of complex trophic interactions (Wang, Wang, Tang, et al., 2018). Linking the ecological indicators of fish communities to human interference remains an important goal for river managers (Zou et al., 2020). The distribution, composition, and diversity of fish communities are commonly used as proxies for assessing an ecosystem's health and integrity. To appropriately manage and protect aquatic ecosystems, it is essential to develop effective and eco‐friendly monitoring approaches to collect field data and obtain biological parameters (Kumar et al., 2022; Shu et al., 2021).

Traditional monitoring of fish diversity has depended largely on census methods such as electrofishing, gill/hoop/seine netting, and dredging/trawling (Wang, Luo, et al., 2020; Wang, Tang, et al., 2019). However, these methods have always been limited by their low sampling efficiency, destructiveness to organisms, and strict reliance on taxonomic expertise (Sakata et al., 2020; Zhang et al., 2020). The application of environmental DNA (eDNA) metabarcoding for fish diversity analysis has emerged and offers a new avenue for characterizing fish diversity in river ecosystems (Pont et al., 2018). This approach provides cost‐effective, dependable, rapid, and continuous investigations for monitoring and assessing fish diversity, which is crucial for the timely and effective management and conservation of river and estuary ecosystems (Garlapati et al., 2019).

The metabarcoding approach coupled with the use of eDNA is a potentially powerful tool for surveying and assessing aquatic diversity. Numerous studies have demonstrated the utility of eDNA metabarcoding for assessing fish diversity (Rourke et al., 2022). Researchers have successfully applied eDNA metabarcoding to monitor fish diversity in freshwater and seawater samples from different habitats, especially in streams, reservoirs, estuaries, and oceans (Civade et al., 2016; Stoeckle et al., 2017; Yao et al., 2022; Zou et al., 2020). The results from these studies have shown that eDNA metabarcoding is a sound biomonitoring tool for use in the conservation and management of aquatic ecosystems (Nguyen et al., 2020). Currently, the application of eDNA metabarcoding for monitoring and assessing biodiversity is at the forefront of the available methods used by ecologists and conservation scientists (Beng & Corlett, 2020; Bernos et al., 2023).

Many studies have compared eDNA results to those from traditional methods and have shown a correlation between the results from these two approaches (Lacoursière‐Roussel et al., 2016; Port et al., 2016). In some studies, eDNA analysis was superior for characterizing fish biodiversity compared to traditional techniques such as electrofishing and hoop netting (Nguyen et al., 2020; Pont et al., 2018). In other studies, the results obtained from eDNA have been comparable to those of traditional methods in which fishes are caught through visual dive surveys and trawling (Port et al., 2016; Zou et al., 2020). Previous studies have shown that, compared with physical collection, eDNA metabarcoding results in greater taxon diversity; however, the practical application of eDNA in evaluating the composition and structure of fish communities has been less explored, and whether eDNA could replace traditional monitoring has not been explored.

The river systems of southern China, in a typical subtropical monsoon climate zone, serve as reserves for biodiversity conservation. Due to the disproportionate use of coastal wetland resources and intense anthropogenic activities (e.g., drainage, reclamation, and pollution), subtropical river ecosystems (e.g., the Pearl River) have been badly damaged, and their biodiversity and bioresources have seriously declined. To investigate the distribution and composition of the fish communities in this area, eDNA metabarcoding studies combined with electrofishing surveys were conducted concurrently. The fish diversities calculated by traditional method (i.e., species abundance and biomass collected by electrofishing) and by eDNA operational taxonomic unit (OTU) richness were compared. The objectives of our study included (1) using eDNA to assess the composition and diversity of fish communities in subtropical rivers, (2) revealing the similarities and differences in fish community characteristics between eDNA and traditional methods, and (3) exploring the application of fish eDNA for evaluating environmental status (e.g., water and habitat quality) in disturbed river systems.

## 2 MATERIALS AND METHODS

### 2.1 Study area and sampling sites

**FIGURE 1:**
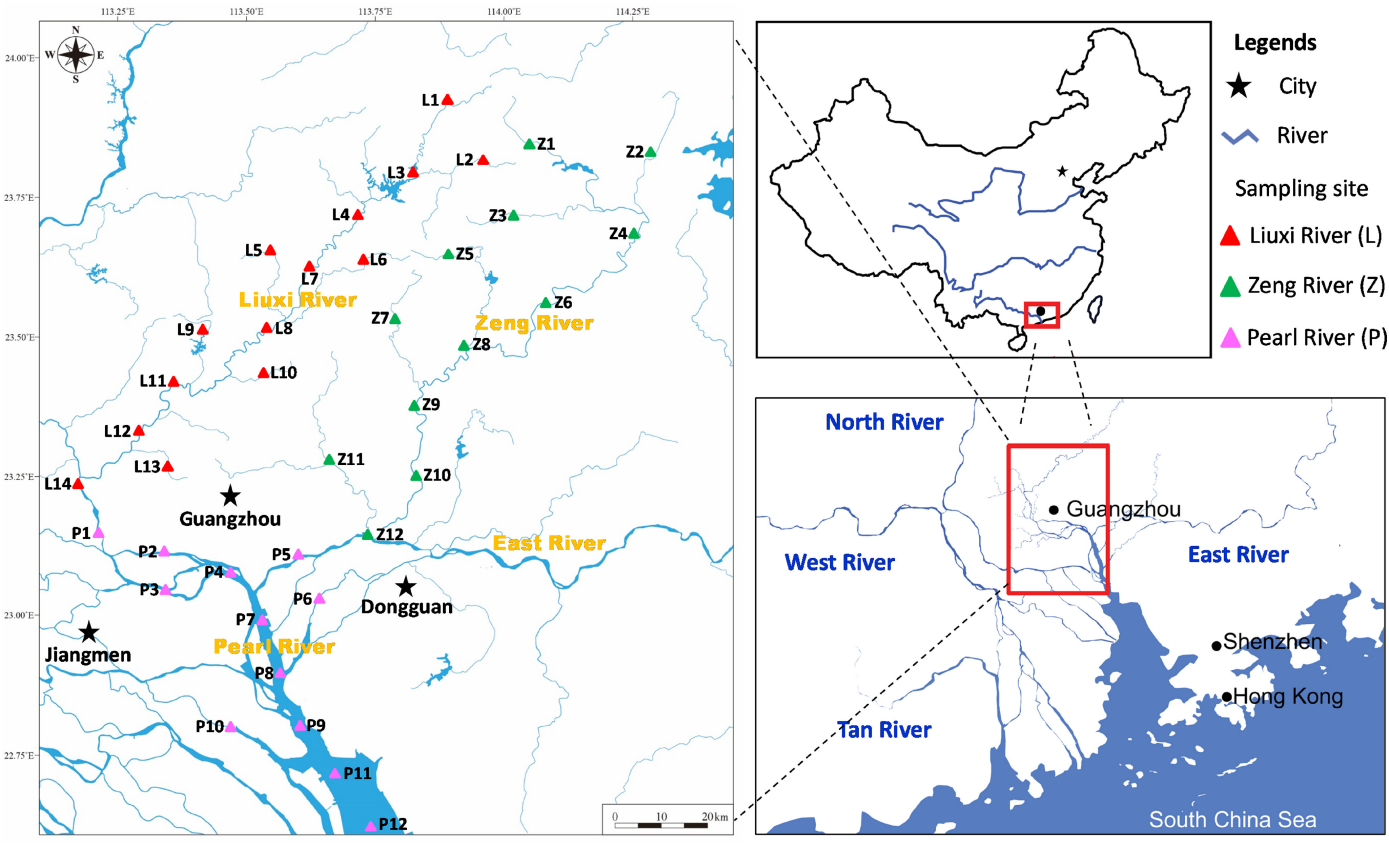
Locations of the 38 sampling sites along the Liuxi River (L1–L14), Zeng River (Z1–Z12), and Pearl River (P1–P12).

The Pearl River, the second largest river in China, is in the tropical and subtropical areas of Southeast Asia and serves as a critical water source for Guangdong Province and Hong Kong. The water sources of the Pearl River are essential for supplying drinking water, generating power, and performing irrigation in Guangdong Province. Thus, the ecological health of the Pearl River is important for the sustainable development of the Pearl River Delta. The Pearl River consists of multiple tributaries—the North River, West River, East River, Liuxi River, and Zeng River—which merge into the Pearl River Estuary in southern China. The current survey originated from the Liuxi River and Zeng River (Figure 1), which are regarded as water sources with high cultural significance for Guangzhou, the capital city of Guangdong Province in southern China. The Liuxi River and Zeng River are upstream source waters for the Pearl River, including both upstream wadable and downstream nonwadable habitats. Thus, these rivers with heterogeneous habitats are important for comparing the eDNA metabarcoding and electrofishing method.

Thirty‐eight sampling sites were chosen from the headwaters of the Liuxi River (L1–L14) and Zeng River (Z1–Z12) to the mainstream of the Pearl River (P1–P12) and then to the Pearl River mouth connected with the South China Sea (Figure 1). The landforms within the drainage basin are dominated by medium–low mountains and hills. The mean annual temperature and precipitation are 21.6°C and 2188 mm, respectively. Approximately, 83.3% of the runoff is discharged during the flood season (from April to September). The zonal soil and vegetation are Udic Ferralisols and southern subtropical evergreen broad‐leaved forest, respectively. The forest coverage is approximately 50%. Arable land accounts for only 8.7% of the drainage area, with paddy fields accounting for most of this area. Environmental data, including physicochemical parameters of water quality, habitat characteristics, and bacterial amounts (Table S1), were provided by two nationally accredited (China Metrology Accreditation) third‐party testing agencies (Hainan Qingxiao Environmental Testing Co., Ltd. and Hainan Qianchao Ecological Technology Co., Ltd.).

### 2.2 Electrofishing sampling for fish investigation

Fish were collected by electrofishing from the headwaters to the estuary along the three rivers (Figure 1) during the rainy season in July 2022. The electrofishing equipment consisted of a 24‐kW generator, a 12 V‐160 A lithium battery, a silicon‐controlled inverter, and two continuously adjustable voltage and frequency regulators. This equipment was used to effectively stun and collect fish (individual weight < 10 kg) in a 1.5‐m‐wide × 1.5‐m‐long × 2.5‐m‐deep water column. Due to varying water levels, two electrofishing operations were conducted following basic guidelines (Barbour et al., 1999; Hauer & Lamberti, 2007). At wadeable sites, single‐pass backpack electrofishing was performed by two operators moving in a zig‐zag fashion, and the walking speed was controlled to ensure a sampling effort of approximately 8 m^2^/min over 30 min. At nonwadeable sites, personnel and equipment were loaded with a welded hull boat, and a bamboo quant was used to propel the boat to eliminate noise disturbance to the fish. Due to the high water depth (1.0–2.5 m), a large scoop net was used by a sternward auxiliary to collect the stunned benthic fish. Boat electrofishing was conducted over 500 m, spanning both riverbanks with a sampling effort of approximately 6 m^2^/min (Flotemersch et al., 2006). The abundance (individual number) and biomass (weight mass) of the fish specimens were collected as traditional fishing data.

### 2.3 Environmental DNA sampling, extraction, and metabarcoding

Sampling for eDNA was also conducted in July 2022, just before seasonal electrofishing occurred in the three rivers. Before electrofishing, three 2.5‐L water samples were collected from each site, one each at the downstream, midstream, and upstream boundaries. We sampled at the most downstream location first and then moved upstream. To avoid disturbing sediment or organisms, we used an extended pole with a dip bucket to collect surface water. The pole and dip bucket were wiped with 10% bleach and rinsed with Milli‐Q water before each collection. During each sampling process, an additional 2.5 L bottle was filled with Milli‐Q water in the laboratory prior to sampling and was transported alongside field sampling bottles to serve as a full‐process negative control.

Within 24 h of collection, the river water and negative control samples were filtered using Whatman glass microfiber filter papers (47 mm diameter, 1.2 μm pore size). DNA from water was extracted using the Qiagen DNeasy Tissue and Blood DNA Extraction Kit following the manufacturer's protocol with minor modifications. Three membranes (1 L of water per membrane) for each sample were cut into pieces, ground and mixed. Then, the sample was soaked in 600 μL of 2 × lysis buffer AL and 40 μL of proteinase K. Incubation with this mixture was performed at 56°C for 2.5 h. Finally, we washed the filters in the mixture and performed elution in 200 μL of AE buffer. Filtration blanks and negative controls were extracted alongside the samples and subjected to the same protocol as the samples. The DNA concentration was determined using a Qubit dsDNA HS Assay Kit and detected on a 1.0% agarose gel. No data or bands were observed for the filtration blanks or negative controls.

Metabarcoding was performed in duplicate on each DNA extract with the primers MiFish‐U‐F (5′GTCGGTAAAACTCGTGCCAGC‐3′) and MiFish‐U‐R (5′‐CATAGTGGGGTATCTAATCCCAGTTTG‐3′), which target the 12S rDNA region (amplifying an ~180 bp region) of the mitochondrial genome, to identify the fish species. Eight‐base barcodes were added to each sample that was destined for sequencing. DNA amplification was performed via a two‐step PCR protocol designed for the BGISEQ‐500 platform. For each set of replications, environmental samples, filtration blanks, and negative controls were included. The PCR assay volume was 50 μL, including 0.3 μL of Takara Ex Taq (5 U/μL), 5 μL of 10 × Ex Taq buffer (20 mM Mg^2+^ plus), 4 μL of a dNTP mixture (10 μM), 1 μL of the forwards and reverse primers (10 μM), 1 μL of the DNA template (environmental samples, filtration blanks and negative controls), and molecular biology‐grade water added to 50 μL. For all the samples, the first‐step PCR was performed as follows: 94°C for 5 min, followed by 10 cycles of 94°C for 30 s, 60°C for 30 s, and 72°C for 30 s, with a final extension at 72°C for 10 min. The first‐step PCR products were diluted five times with molecular biology‐grade water and used as templates for the second‐step PCR. For subsequent sequencing on the BGISEQ‐500 platform, two or three random nucleotides were inserted into the MiFish primers (to increase sequence diversity during sequencing). The second‐step PCR was carried out in a 50 μL reaction volume including 0.3 μL of Takara Ex Taq (5 U/μL), 5 μL of 10× Ex Taq buffer (20 mM Mg^2+^ plus), 4 μL of a dNTP mixture (10 μM), 1 μL of the forwards and reverse primers with the BGISEQ‐500 adapter (10 μM), 2 μL of the template, and molecular biology‐grade water added to 50 μL. The PCR procedure was as follows: 94°C for 5 min, followed by 20 cycles of 94°C for 30 s, 60°C for 30 s, and 72°C for 30 s, with a final extension at 72°C for 10 min. After the two‐step PCR amplification, the PCR products were detected on a 1.5% agarose gel. None of the filtration blanks or negative controls showed amplification.

### 2.4 Library construction and Illumina sequencing

The PCR products showing the target bands were mixed in equal amounts, followed by electrophoresis in a 2% agarose gel and gel cutting. The PCR products were purified using a QIAquick Gel Extraction Kit. Sixty nanograms of the purified PCR products were denatured at 95°C and ligated with T4 DNA ligase at 37°C to generate a single‐stranded circular DNA library. Library replicates and negative controls were simultaneously included throughout the process. The concentrations and fragment size distributions of the libraries were checked on an Agilent 2100 Bioanalyzer. All the libraries were subsequently pooled in equal amounts to generate DNA nanoballs (DNBs). Each DNB was pooled into 1 lane for sequencing. Sequencing was performed via 150 bp paired‐end sequencing on the BGISEQ‐500 high‐throughput platform. Then, paired‐end reads were combined with tags based on overlaps using FLASH (Magoč & Salzberg, 2011). The tags were clustered into OTUs using USEARCH with a 97% threshold, and chimera were filtered out using UCHIME (Rognes et al., 2016). All tags were mapped to each representative OTU sequence using USEARCH to construct the OTU richness table. The taxonomic assignment of OTU sequences was mapped to the NT database downloaded from the National Center for Biotechnology Information (NCBI) GenBank database using the Blastn tool. Sequences were considered to belong to a species if they had ≥96% sequence identity to the NT and NCBI database barcodes across the entire length of the amplicon. If a sequence could be assigned to several species (≥99% matching rate) and the species belonged to the same genus, the taxonomic resolution collapsed to the genus level. Rarefaction curves based on the number of reads against the number of OTUs were provided in Figure S1 of the SI to show that the analysis has been carried out at a sufficient sequence depth.

### 2.5 Contamination analysis and data censoring

We compared the distribution of taxon abundances in the negative controls with that in the whole river water sample dataset. Most taxa were either absent from negative controls or present at a rate that scaled log‐linearly with total counts, asymptotically approaching an occurrence rate of approximately 0.01%, similar to what has been reported elsewhere (Olds et al., 2016). Eight taxa present in the negative controls (Table S2), including exotic (*Salmo salar*, *Salvelinus fontinalis*, *Rhabdosargus haffara*, *Photopectoralis bindus*, and *Osmerus mordax*) and oceanic species (*Doratonotus megalepis*, *Hipposcarus harid*, and *Epinephelus fuscoguttatus* commonly found in coral reefs), have never been recorded in the three rivers. The strongest instances of contamination by these taxa were due to the routine use of fish specimens during other PCR experiments. The excess reads of these taxa were generally associated with two of the negative control samples. This pattern indicated point occurrence than systematic contamination. Given that these contamination events impacted single control samples, it was impossible to determine which biological samples, if any, were also contaminated. Nonetheless, for the purpose of assessing taxon and sample correlations, sample richness, and other comparative analyses, we removed all contaminant taxa and the species with zero or negative OTU reads (river water samples minus negative controls). All raw sequences have been stored in the public NCBI Sequence Read Archive database under the BioProject number PRJNA1080492 (http://www.ncbi.nlm.nih.gov/sra/PRJNA1080492).

### 2.6 Data processing and statistical analysis

All the statistical analyses were conducted in R 4.2.3 with the following packages: *cluster*, *factoextra*, *phyloseq*, *vegan*, and *ggplot2*. Nonmetric multidimensional scaling (NMDS) was used to group the 38 sampling sites into spatial zones with distinct differences in fish community structure according to the relative composition (%) of OTU richness, species abundance, and biomass. NMDS relies on the rank order of pairwise variable dissimilarities (Euclidean distance in this study) and does not make any underlying distributional assumptions about the data (Borcard et al., 2011). The sampling sites were plotted in ordination space with the distance between points positively related to the dissimilarity of the output parameters (i.e., sites with similar output parameters were plotted closer to one another). The analysis of similarity (ANOSIM) test was used to evaluate the dissimilarity matrix and test whether groups of objects had significantly (*p* < .05) different mean dissimilarities.

Based on the OTU richness, the pairwise taxonomic Bray–Curtis dissimilarity matrix between different samples was calculated using the *microeco* package (Liu et al., 2021). The values of environmental factors and fish OTU richness that exhibited significant variations were used, and stepwise forward selection was performed to linearly reduce the number of correlated variables along the axes. A permutation limit (with a *p* value of .05) was used to determine which variables should be incorporated into the final model. The relationships between the eDNA‐based and abundance−/biomass‐based Shannon indices of alpha diversity were estimated via linear regression. Linear dependencies were explored by computing the variable variance inflation factors to ensure no confounding collinearity. The statistical significance of the axes derived from each analysis was tested with a Monte Carlo test (999 permutations).

Linear discriminant analysis effect size (LEfSe) is an algorithm used for discovering high‐dimensional indicators that identify taxa by characterizing the differences between two or more biological conditions (Segata et al., 2011). LEfSe emphasizes both statistical significance and biological relevance, enabling researchers to identify discriminative features that are significantly different between biological classes. The nonparametric factorial Kruskal−Wallis sum‐rank test was first used to detect features with significant differential abundance with respect to the class of interest. Second, LEfSe uses linear discriminant analysis to estimate the effect size of each differentially abundant feature and rank the feature accordingly (Liu et al., 2021).

Two‐way hierarchical clustering analysis was performed using the *pheatmap* package. The package used clustering distances and methods implemented in the *dist* and *hclust* functions in R. The clustering analysis placed fish species with similar responses to the environmental factors into a group. Statistically significant cluster trees were identified using a bootstrap randomization technique in which the nonzero values were resampled and used to generate pseudovalues under the null hypothesis. The results are displayed as a heatmap.

## 3 RESULTS

### 3.1 Spatial distribution patterns of fish communities along the river

**FIGURE 2:**
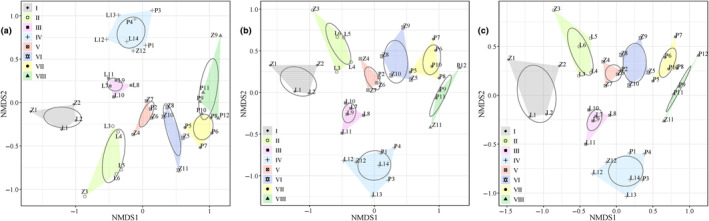
Eight spatial zones (I–VIII) grouped by NMDS based on the relative percentage (%) of OTU richness (a), species abundance (b), and biomass (c) of the fish communities at the 38 sampling sites (L1–L14 in the Liuxi River, Z1–Z12 in the Zeng River, and P1–P12 in the Pearl River).

There were 127 fish species belonging to 19 orders, 54 families, and 99 genera detected by the eDNA sampling protocol. There were 88 fish species belonging to 16 orders, 43 families, and 75 genera detected by electrofishing in situ. The fish species detected by eDNA across the 38 sampling sites included all the fish species sampled by electrofishing, indicating that the capacity of eDNA to detect species was greater than that of electrofishing. NMDS results showed that, regardless of the data type (i.e., eDNA OTU richness/reads and traditional observation counting), the relative percentage (%) of OTU reads (Figure 2a and Tables S3 and S4), species abundance (Figure 2b and Table S5), and biomass (Figure 2c and Table S6) could be used to group the 38 sampling sites into eight spatial zones, namely, I (L1−L2 and Z1–Z2), II (L3−L6 and Z3), III (L7−L11), IV (L12−L14, Z12, P1, and P3−P4), V (Z4, Z6–Z7, and P2), VI (Z5, Z8, and Z10−Z11), VII (P5−P8 and P10), and VIII (Z9, P9, and P11−P12). Along the river continuum, these zones were distributed in sections with heterogeneous habitats, including headwaters, upper streams, middle rivers, lower reaches, and river mouths. Our results suggested that eDNA could indicate the spatial distribution pattern of fish communities as well as the pattern reflected by electrofishing.

### 3.2 The composition of fish communities at different taxonomic levels

**FIGURE 3:**
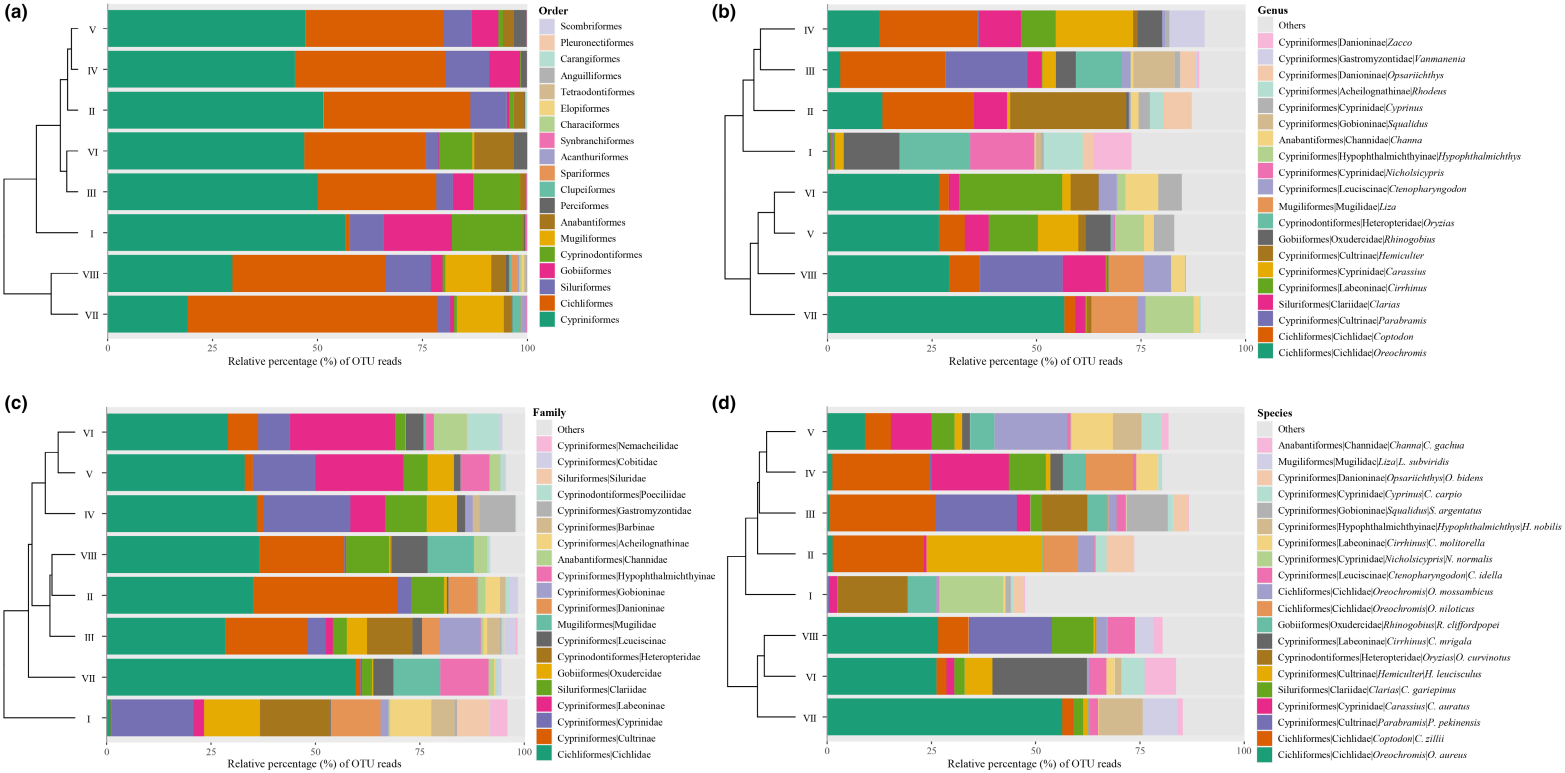
The composition of fish communities and the clustering of spatial zones based on the relative percentage (%) of OTU reads. The first 20 dominant orders, families, genera, and species are shown in (a), (b), (c), and (d), respectively.

The relative percentage of eDNA OTU richness showed that, at the order level (Figure 3a), Cypriniformes, Cichliformes, Siluriformes, and Gobiiformes made up >83% of the fish communities in the three rivers. At the family level (Figure 3b), Cichlidae, Cultrinae, Cyprinidae, and Labeoninae made up >56% of the fish communities in Zones II−VIII, whereas Cyprinidae, Oxudercidae, and Heteropteridae made up >50% of the fish communities in Zone I. At the genus (Figure 3c) and species levels (Figure 3d), the composition of the fish communities was dispersed and determined by different genera and species. Notably, except for zones I and II, where native *Oryzias curvinotus* and *Hemiculter leucisculus* had the highest OTU richness, respectively, exotic *Coptodon zillii* and *Oreochromis aureus* had the highest OTU richness in the other zones, indicating that the structure of the fish communities in the middle and lower reaches was dominated by alien species. These results indicated that eDNA metabarcoding has advantages in identifying dominant species and exotic species.

A cluster analysis of the eight spatial zones showed that the order‐ and family‐level groupings (Figure 3a,b) were insufficient to distinguish the longitudinal differences in the fish community composition from the headwaters to the estuary. Compared with the order‐ and family‐level groupings, the genus‐level (Figure 3c) grouping could more clearly distinguish the differences between upstream zones I−IV and downstream zones V−VIII. Moreover, species‐level grouping (Figure 3d) was the most effective at distinguishing upstream zones I−III, midstream zones IV−V, and downstream zones VI–VIII. Interestingly, at the family level, upstream zone II and downstream zone VIII were clustered in the same group, which was caused by the widespread species of Cichlidae, Cultrinae, and Oxudercidae that could not be distinguished by family‐level identification. In contrast, compared with those at the family level, the higher resolutions at the genus and species levels could distinguish *Coptodon* and *Oreochromis* of Cichlidae as well as *Hemiculter* and *Parabramis* of Cultrinae, which were the key genera that distinguished the fish distributions in the upper and lower reaches.

### 3.3 Diversity relationships between eDNA and electrofishing samples

**FIGURE 4:**
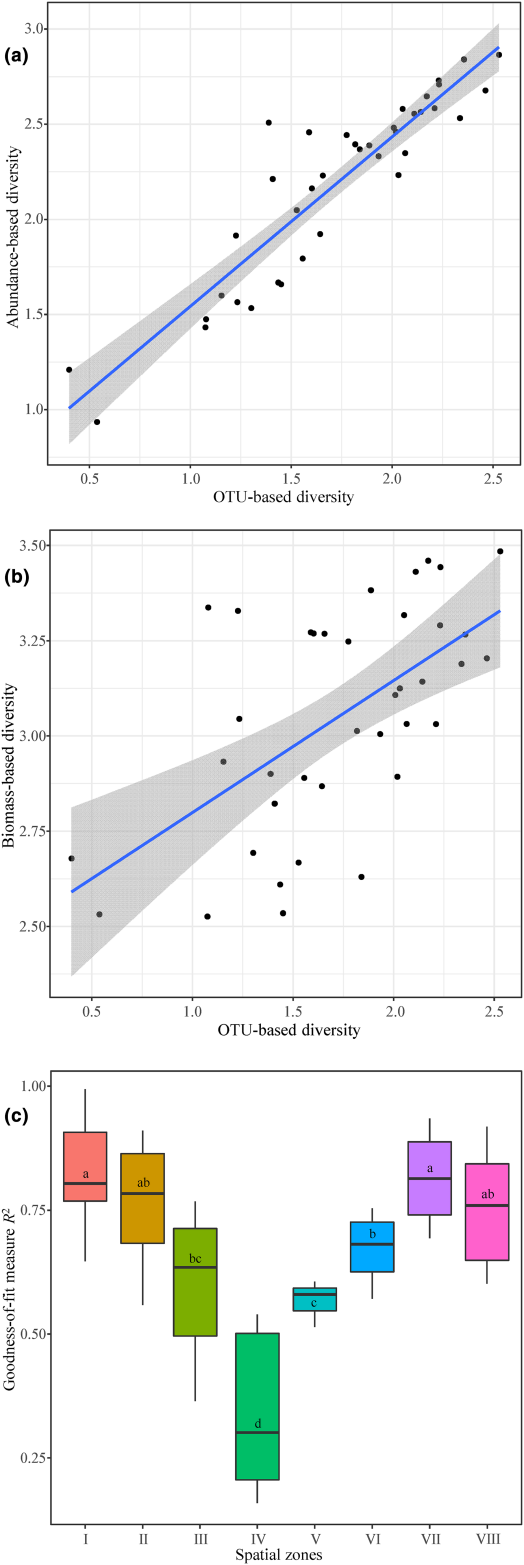
Linear regression between the alpha diversity (Shannon index) calculated by the eDNA (i.e., OTU richness and reads) and electrofishing (i.e., species abundance and biomass) methods. (a) Linear regression between OTU‐ and abundance‐based diversity. (b) Linear regression between OTU‐ and biomass‐based diversity. (c) Spatial variation in the *R* ^2^ of linear regression between OTU‐ and abundance‐based diversity. Different superscript letters represent significant (*p* < .05) differences.

The linear regression results showed that the relationships between the alpha diversity (Shannon index) calculated by the OTU reads and the fish abundance exhibited significant (*p <* .05) positive correlations (Figure 4a). The regression equation was fitted as *Y* = 0.9572∙*X* + 0.5425 (*R* ^2^ = .8218), where *Y* is the abundance‐based diversity, *X* is the OTU‐based diversity, and *R*‐squared (*R* ^2^) measures how close the data points are to the fitted line. The linear regression between the alpha diversity calculated by the OTU reads and the fish biomass (wet weight) exhibited significant (*p <* .05) positive correlations. The regression equation was fitted as *Y* = 0.4339∙*X* + 2.3043 (*R* ^2^ = .4558), where *Y* is the biomass‐based diversity and *X* is the OTU‐based diversity (Figure 4b). These results indicated that eDNA metabarcoding could reflect the diversity of local fish communities as well as the diversity determined by traditional observation counting; thereinto, the OTU‐based diversity had a greater degree of match with abundance‐based diversity than with biomass‐based diversity. In addition, *R* ^2^ showed significant (*p <* .05) intergroup differences between the spatial zones (Figure 4c). The highest *R* ^2^ values were observed in zones I−II and VII–VIII, which were significantly greater than those in zones IV−V. These results indicated that the OTU‐based diversity had greater linear regression with abundance‐based diversity in the headwater and estuarial zones.

### 3.4 Biomarkers that characterize site‐specific fish community structure

**FIGURE 5:**
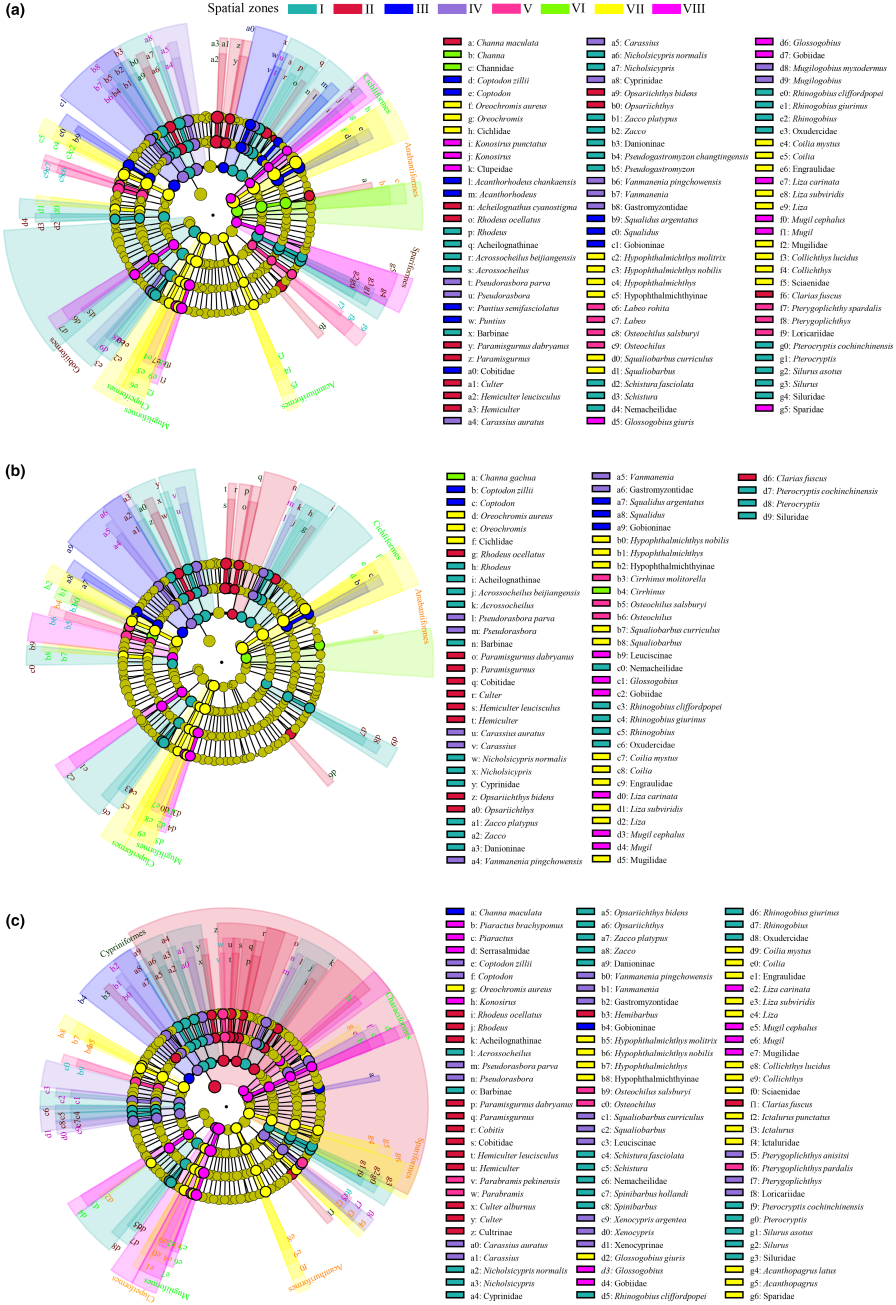
Fish biomarkers selected by LEfSe from the eight spatial zones in the three subtropical rivers. (a) Biomarkers selected by LEfSe with relative percentage (%) of OTU reads. (b) Biomarkers selected by LEfSe with relative percentage (%) of species abundance. (c) Biomarkers selected by LEfSe with relative percentage (%) of species biomass. The circles represent the classification level of order, family, genus, and species from the outside to the inside; the size of the fan‐shaped area is proportional to the representativeness of the fish biomarkers.

OTU‐based LEfSe selected 6 orders, 20 families, 34 genera, and 38 species as biomarkers (Figure 5a). Abundance‐based LEfSe selected 4 orders, 18 families, 22 genera, and 26 species as biomarkers (Figure 5b). Biomass‐based LEfSe selected 6 orders, 22 families, 34 genera, and 37 species as biomarkers (Figure 5c). The common biomarkers (Table 1) selected by LEfSe from eDNA and electrofishing methods were Acheilognathinae*|Rhodeus*, Barbinae|*Acrossocheilus*|*A. beijiangensis*, Nemacheilidae|*Schistura*|*S. fasciolata*, *Nicholsicypris*|*N. normalis*, Danioninae|*Zacco*|*Z. platypus*, Siluridae*|*(*Silurus|S. asotus* and *Pterocryptis|P. cochinchinensis*), and Oxudercidae|*Rhinogobius*|(*R. giurinusi* and *R. cliffordpopei*) in zone I; *Rhodeus ocellatus*, *Culter*, *Hemiculter*|*H. leucisculus*, *Opsariichthys*|*O. bidens*, *Paramisgurnus*|*P. dabryanus*, and *Clarias fuscus* in zone II; Gobioninae|*Squalidus*|*S. argentatus* and *Coptodon*|*C. zillii* in zone III; *Carassius*|*C. auratus*, Gastromyzontidae|*Vanmanenia*|*V. pingchowensis*, and *Pseudorasbora*|*P. parva* in zone IV; *Pterygoplichthys pardalis* and *Osteochilus*|*O. salsburyi* in zone V; Hypophthalmichthyinae|*Hypophthalmichthys*|(*H. nobilis* and *H. molitrix*), Mugilidae|*Liza*|*L. subviridis*, Cichlidae|*Oreochromis*|*O. aureus*, Engraulidae|*Coilia*|*C. mystus*, Sciaenidae|*Collichthys*|*C. lucidus*, and *Squaliobarbus*|*S. curriculus* in zone VII; *Konosirus*, Gobiidae|*Glossogobius*, *Mugil*|*M. cephalus*, and *Liza carinata* in zone VIII. Notably, no common biomarkers were found in zone VI, indicating that the fish community structure characterized by biomarkers differed greatly between eDNA and electrofishing methods in this area.

**TABLE 1:** Common fish biomarkers selected by LEfSe through three data types: eDNA OTU richness (superscript E), species abundance (subscript A), and biomass (subscript B).

Zone	(Sub)family	Genus	Species	
I	${\text{Acheilozgnathinae}}_{A}^{E}$	${\text{Rhodeus}}_{A}^{E}$	—	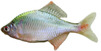
	${\text{Barbinae}}_{A+B}^{E}$	${\text{Acrossocheilus}}_{A+B}^{E}$	$A.\;{\text{beijiangensis}}_{A}^{E}$	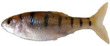
		*Spinibarbus*_B_	*S. hollandi*_B_	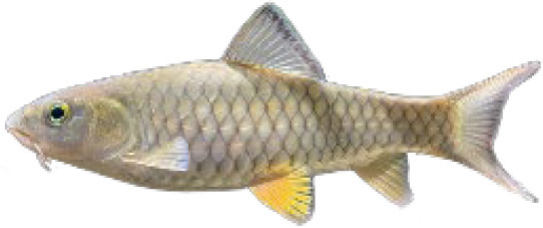
	${\text{Nemacheilidae}}_{A+B}^{E}$	${\text{Schistura}}_{B}^{E}$	$S.\;{\text{fasciolata}}_{B}^{E}$	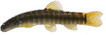
	Cyprinidae_A+B_	${\text{Nicholsicypris}}_{A+B}^{E}$	$N.{\text{normalize}}_{A+B}^{E}$	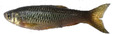
	—	*Pseudogastromyzon* ^E^	*P. changtingensis* ^E^	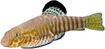
	${\text{Danioninae}}_{A+B}^{E}$	${\text{Zacco}}_{A+B}^{E}$	$Z.\;{\text{platypus}}_{A+B}^{E}$	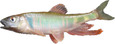
		*Opsariichthys*_B_	*O. bidens*_B_	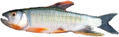
	${\text{Siluridae}}_{A+B}^{E}$	${\text{Silurus}}_{B}^{E}$	$S.\;{\text{asotus}}_{B}^{E}$	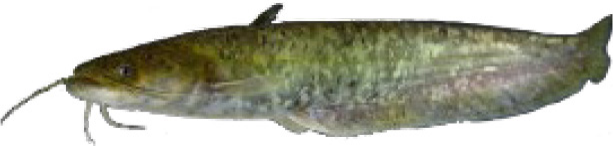
		${\text{Pterocryptis}}_{A+B}^{E}$	$P.\;{\text{cochinchinensis}}_{A+B}^{E}$	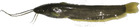
	${\text{Oxudercidae}}_{A+B}^{E}$	${\text{Rhinogobius}}_{A+B}^{E}$	$R.{\text{giurinus}}_{A+B}^{E}$	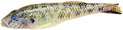
			$R.\;{\text{cliffordpopei}}_{A+B}^{E}$	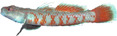
II	Cobitidae_A+B_	*Cobitis*_B_	—	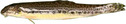
	Acheilognathinae_B_	*Rhodeus*_B_	$\text{Rhodeus}\;{\text{ocellatus}}_{A+B}^{E}$	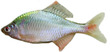
		—	*Acheilognathus cyanostigma* ^E^	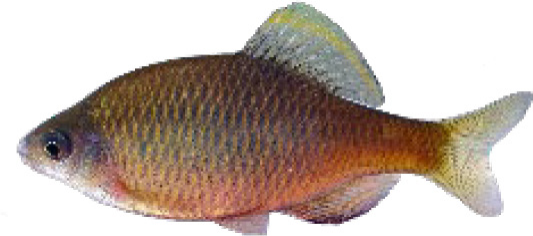
	Cultrinae_B_	${\text{Culter}}_{A+B}^{E}$	*C. alburnus*_B_	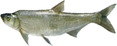
		${\text{Hemiculter}}_{A+B}^{E}$	$H.\;{\text{leucisculus}}_{A+B}^{E}$	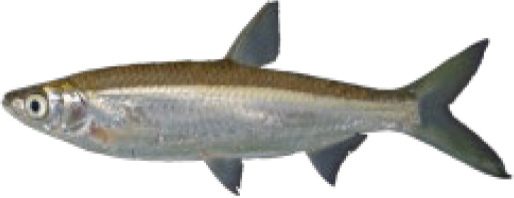

	—	*Hemibarbus*_B_	—	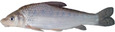
	—	${\text{Opsariichthys}}_{A}^{E}$	$O.\;{\text{bidens}}_{A}^{E}$	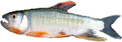
	—	${\text{Paramisgurnus}}_{A+B}^{E}$	$P.{\text{dabryanus}}_{A+B}^{E}$	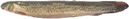
	—	—	$\text{Clarias}\;{\text{fuscus}}_{A+B}^{E}$	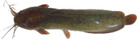
—	—	*Channa maculata* ^E^	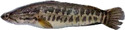
III	Cobitidae^E^	—	—	
	${\text{Gobioninae}}_{A+B}^{E}$	${\text{Squalidus}}_{A}^{E}$	$S.\;{\text{argentatus}}_{A}^{E}$	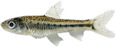
	—	${\text{Coptodon}}_{A}^{E}$	$C.\;{\text{zillii}}_{A}^{E^{*}}$	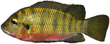
	—	*Acanthorhodeus* ^E^	*A. chankaensis* ^E^	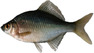
	—	*Puntius* ^E^	*P. semifasciolatus* ^E^	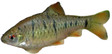
	—	—	*Channa maculata*_B_	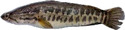
IV	Cyprinidae^E^	${\text{Carassius}}_{A+B}^{E}$	$C.\;{\text{auratus}}_{A+B}^{E}$	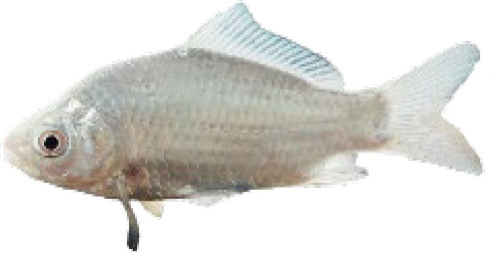
	${\text{Gastromyzontidae}}_{A+B}^{E}$	${\text{Vanmanenia}}_{A+B}^{E}$	$V.\;{\text{pingchowensis}}_{A+B}^{E}$	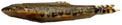
	Xenocyprinae_B_	*Xenocypris*_B_	*X. argentea*_B_	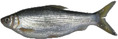
	Leuciscinae_B_	*Squaliobarbus*_B_	*S. curriculus*_B_	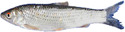
	Loricariidae_B_	*Pterygoplichthys*_B_	$P.\;{\text{anisitsi}}_{B}^{*}$	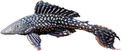
	—	${\text{Pseudorasbora}}_{A+B}^{E}$	$P.\;{\text{parva}}_{A+B}^{E}$	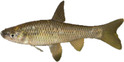

	—	*Mugilogobius* ^E^	*M. myxodermus* ^E^	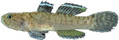
	*Coptodon*_B_	$C.\;{\text{zillii}}_{B}^{*}$	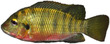
V	Loricariidae^E^	*Pterygoplichthys* ^E^	$P.\;{\text{pardalis}}_{B}^{E^{*}}$	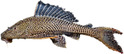
	—	${\text{Osteochilus}}_{A+B}^{E}$	$O.\;{\text{salsburyi}}_{A+B}^{E}$	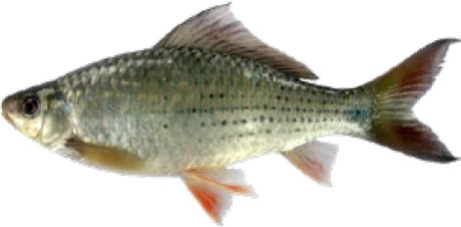
	—	*Labeo* ^E^	$L.\;{\text{rohita}}^{E^{*}}$	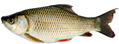
	—	—	*C. molitorella*_A_	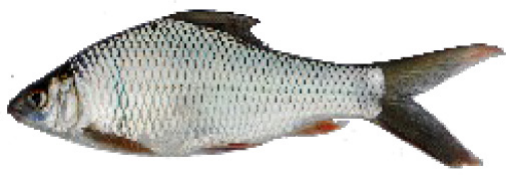
	—	*Parabramis*_B_	*P*. *pekinensis*_B_	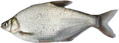
VI	Channidae^E^	*Channa* ^E^	*Channa gachua*_A_	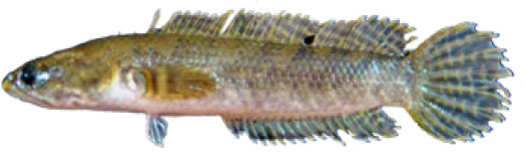
	—	*Cirrhinus*_A_	—	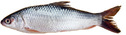
VII	${\text{Hypophthalmichthyinae}}_{A+B}^{E}$	${\text{Hypophthalmichthys}}_{A+B}^{E}$	$H.\;{\text{nobilis}}_{A+B}^{E}$	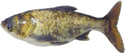
			$H.\;{\text{molitrix}}_{B}^{E}$	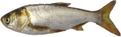
	${\text{Mugilidae}}_{A}^{E}$	${\text{Liza}}_{A+B}^{E}$	$L.{\text{subviridis}}_{A+B}^{E}$	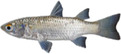
	${\text{Cichlidae}}_{A}^{E}$	${\text{Oreochromis}}_{A}^{E}$	$O.\;{\text{aureus}}_{A+B}^{E^{*}}$	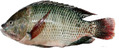
	Ictaluridae_B_	*Ictalurus*_B_	$I.{\text{punctatus}}_{B}^{*}$	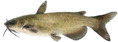
	Sparidae_B_	*Acanthopagrus*_B_	*A. latus*_B_	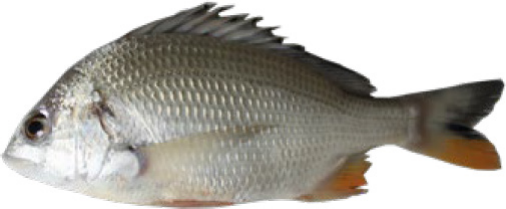
	${\text{Engraulidae}}_{A+B}^{E}$	${\text{Coilia}}_{A+B}^{E}$	$C.\;{\text{mystus}}_{A+B}^{E}$	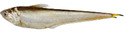

	${\text{Sciaenidae}}_{B}^{E}$	${\text{Collichthys}}_{B}^{E}$	$C.\;{\text{lucidus}}_{B}^{E}$	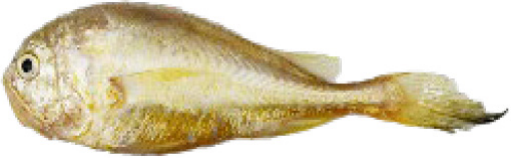
	—	${\text{Squaliobarbus}}_{A}^{E}$	$S.\;{\text{curriculus}}_{A}^{E}$	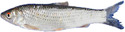
—	—	*Glossogobius giuris*_B_	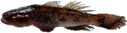
VIII	Sparidae^E^	*—*	*—*	
	Leuciscinae_A_	*—*	*—*	
	Serrasalmidae_B_	*Piaractus*_B_	$P.\;{\text{brachypomus}}_{B}^{*}$	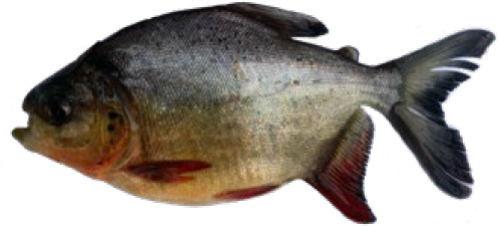
	Clupeidae^E^	${\text{Konosirus}}_{B}^{E}$	*K. punctatus* ^E^	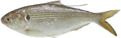
	${\text{Gobiidae}}_{A+B}^{E}$	${\text{Glossogobius}}_{A+B}^{E}$	*G. giuris* ^E^	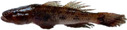
	Mugilidae_B_	${\text{Mugil}}_{A+B}^{E}$	$M.\;{\text{cephalus}}_{A+B}^{E}$	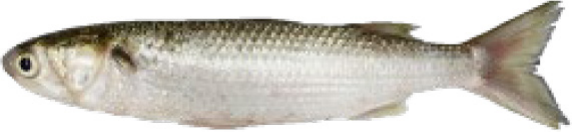
	*—*	*—*	$\text{Liza}\;{\text{carinata}}_{A+B}^{E}$	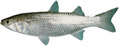

*Note*: If a fish species (e.g., *Nicholsicypris normalis*) can be selected as a biomarker by all three data types, it is marked with “
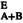
” (e.g., *N. normalize*_A+B_). “—” means no biomarker is selected. “*” indicates exotic species.

Interestingly, the eDNA‐based biomarkers were similar to the abundance‐based biomarkers but differed from the biomass‐based biomarkers. Biomass‐based biomarkers that were not detected by eDNA were bottom‐dwelling species with large body sizes, such as *Spinibarbus*|*S. hollandi* in zone I, Xenocyprinae|*Xenocypris*|*X. argentea* and Loricariidae|*Pterygoplichthys*|*P. anisitsi* in zone IV, *Parabramis*|*P. pekinensis* in zone V, Ictaluridae|*Ictalurus*|*I. punctatus* and Sparidae|*Acanthopagrus*|*A. latus* in zone VII, and Serrasalmidae|*Piaractus*|*P. brachypomus* in zone VIII. Another finding was that the family‐level biomarkers appeared more often in headwater zone I and downstream zones VII−VIII than in other zones, indicating a habitat‐specific distribution of these family‐level biomarkers. For example, in zone I, Barbinae|*Acrossocheilus*, Danioninae|*Zacco*, Siluridae|*Silurus*, and Oxudercidae|*Rhinogobius* are typical rheophilic fish that live in rapids and riffles with cobble substrates. In zone VII, Hypophthalmichthyinae|*Hypophthalmichthys*, Engraulidae|*Coilia*, Cichlidae|*Oreochromis*, and *Squaliobarbus*|*S. curriculus* preferred slow‐flowing and open water bodies. In zone VIII, the appearance of Clupeidae|*Konosirus*, Gobiidae|*Glossogobius*, and Mugilidae|(*Mugil* and *Liza*) indicated brackish water near the estuary. Moreover, *C. zillii* in zone III, *P. pardalis* and *Labeo rohita* in zone V, *O. aureus* in zone VII, and *P. brachypomus* in zone VIII were typical invasive species that could be selected as biomarkers, indicating the sensitivity of eDNA for detecting invasive species.

### 3.5 Relationships between environmental factors and fish community composition

**FIGURE 6:**
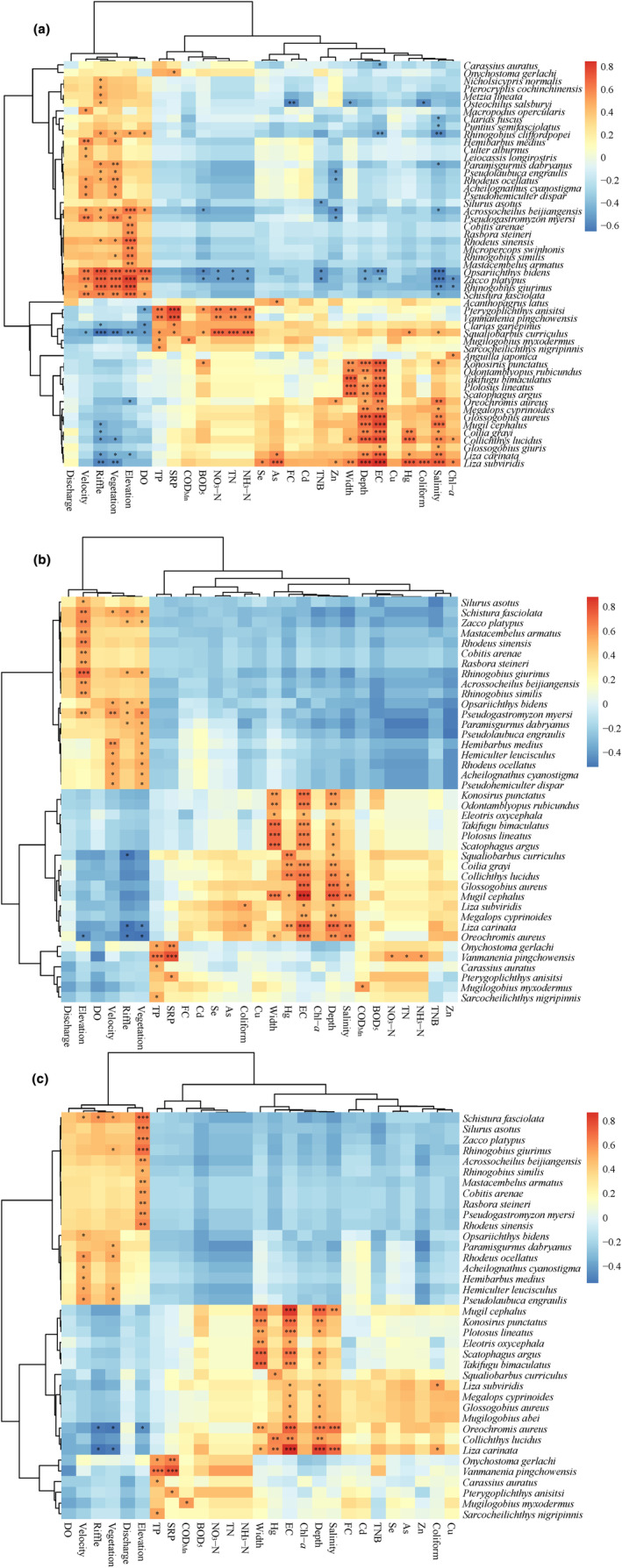
Heatmap of correlations between environmental factors and fish community composition at each sampling site. Correlation coefficients are illustrated using progressive colors from blue (lower values) to red (higher values). Cluster analysis of the fish species and environmental factors based on the correlation matrix are listed on the left and top of the graph, respectively. (a) Correlation heatmap based on environmental factors and relative percentage (%) of OTU reads. (b) Correlation heatmap based on environmental factors and relative percentage (%) of species abundance. (c) Heatmap based on environmental factors and relative percentage (%) of species biomass. Water depth (m); Channel width (m); Flow velocity (m/s); Discharge (m^3^/s); Elevation (m), Riffle area (%); Vegetation cover (%); FC, fecal coliform (MPN/L, most likely number per liter); TNB, total number of bacteria (CFU/100 mL, colony‐forming units per 100 milliliter); DO, dissolved oxygen (mg/L); EC, electrical conductivity (μS/cm); COD_Mn_, chemical oxygen demand determined by Mn (mg/L); BOD_5_, 5‐day biochemical oxygen demand (mg/L); TN, total nitrogen (mg/L); NH_3_−N, ammonia nitrogen (mg/L); NO_3_−N, nitrate nitrogen (mg/L); TP, total phosphorus (mg/L); SRP, soluble reactive phosphorus (mg/L); Chl‐*a*, chlorophyll‐*a* (mg/L); Salinity (‰); and Heavy metal (μg/L), including Cu, Zn, Se, As, Hg, and Cd. “*”, “**”, and “***” indicate significant differences at *p* < .05, *p* < .01, and *p* < .001, respectively.

Correlation analysis between the environmental factors (Table S1) and the composition of the fish communities revealed that 53 (Figure 6a), 40 (Figure 6b), and 38 (Figure 6c) species had significant relationships with the environmental factors according to the relative percentage (%) of the OTU reads, species abundance, and biomass, respectively. In addition, for the eDNA‐based fish community composition, at the order, family, and genus levels, there were 11, 29, and 45 units, respectively, that were significantly (*p <* .05) correlated with the environmental factors (Figure S2). This result indicated that the species‐level identification of the fish community compositions had the strongest response to the environmental changes, followed by genus‐level identification. Interestingly, regardless of the input data type, two environmental gradients did significantly influence the longitudinal distribution of the fish communities. The first environmental gradient was mostly composed of physical water parameters and habitat factors, including elevation (m), dissolved oxygen (mg/L), flow velocity (m/s), riffle area (%), and vegetation cover (%). The high values of these factors indicated pristine habitats in the headwaters and upper reaches, where there was less human disturbance. The second environmental gradient was mostly composed of chemical water parameters (e.g., high COD_Mn_ [mg/L] and BOD_5_ [mg/L]), conductivity (μs/cm), bacterial amounts (total number of bacteria and coliform), and heavy metals (e.g., Hg, As, and Cu [mg/L]). The high values of these factors indicated water pollution and extraneous interference commonly occurring in urban and industrial areas of the lower reaches. These results indicated that eDNA‐based analysis of fish community composition could reflect longitudinal changes in environmental factors along the river, which was similar to the pattern reflected by traditional method (i.e., species abundance and biomass).

## 4 DISCUSSION

The application of nondestructive techniques for monitoring and characterizing fish diversity has drawn increasing worldwide attention for assessing aquatic ecosystem health. Given that investigating fish diversity in large rivers is difficult and costly, finding a new monitoring method with applicable technology has drawn increased attention. The negative impacts of increasing anthropogenic activities on aquatic organisms, especially fish, have been verified in the Pearl River and other rivers and reservoirs connected with it (Wang, Su, et al., 2020; Wang, Tang, et al., 2019; Wang, Wang, et al., 2019; Wang, Wang, Chang, et al., 2018; Wang, Wang, Lin, et al., 2021). To protect and maintain the health of subtropical river systems, we investigated fish diversity along a river continuum using a combination of electrofishing and eDNA metabarcoding. Our results provide evidence that eDNA can reveal the diversity, structure, and biomarkers of fish communities as well as physical collection (e.g., electrofishing). We suggest that researchers and managers use eDNA as an ecological tool to identify fish community structure and associated environmental factors.

### 4.1 The eDNA‐based distribution and composition of fish communities in rivers

The fish species detected by eDNA across the 38 sampling sites included all the fish species sampled by electrofishing, indicating that the capacity of eDNA to find species was greater than that of electrofishing (also see Balasingham et al., 2018; Beng & Corlett, 2020). Generally, the additional species detected by eDNA were the species that have been reported in the Pearl River system but were not distributed in the designed sampling sites of this study. This indicated that, on the one hand, eDNA could trace the DNA released by fish species that were difficult to sample by traditional methods; on the other hand, the DNA of these hard‐to‐catch or rare fish species might be carried by upstream or tributary waters and then collected at downstream or mainstream sites. Although eDNA in water reflects more information on species distributions than the traditional method does, it contains more extraneous species information than traditional in situ field monitoring, which confuses site‐specific assessments of ecological health or biological integrity. In fact, the similar spatial distribution patterns shown by NMDS and the high regression coefficient between OTU‐ and abundance‐based diversity indicated that eDNA could reveal the core information on the composition and diversity of fish communities as comprehensively as that revealed by species abundance and biomass collected by physical in situ investigation. In particular, eDNA‐based biological monitoring for aquatic organisms, including fish and invertebrates, exhibited the highest applicability and accuracy in the wadable streams of the upper reaches and tributaries, where fast‐flowing water facilitates the collection of DNA signals from different fish species.

### 4.2 Appropriate thresholds of taxonomic levels for assessing fish diversity

Compared with aquatic invertebrates, fish have more comprehensive DNA information in the gene bank; thus, the use of eDNA metabarcoding to distinguish fish diversity has broad application prospects. Wang et al. (2023) suggested that in complex habitats, such as fresh and brackish converging lakes, analyzing the invertebrate composition or diversity at the phylum or class level (at least at the family level) is effective enough to reflect environmental properties. The great difference between the usefulness of eDNA for fish and invertebrates is the structure of the primary taxonomic units, which is much simpler for fish than for invertebrates. At the class or order level, different invertebrate assemblages had completely different ecological properties (e.g., freshwater vs brackish or littoral vs limnetic); however, for fishes, order‐level identification could not distinguish habitat‐specific ecological properties. Thus, according to our results, we recommend that an appropriate threshold for eDNA‐based fish monitoring is at the genus or species level, which could be further chosen according to the monitoring or research targets. For example, if users focus on the influence of environmental changes on fish community structure, genus‐level identification with eDNA is effective; if users focus on the spatial distribution of fish assemblages, species‐level identification might be favorable.

### 4.3 Biomarkers that can distinguish the differences between fish communities

The LEfSe results showed that biomarkers selected by both eDNA and electrofishing exhibited similarities in the headwaters and lower reaches but differences in the middle reaches. eDNA could be used to distinguish fishes of Danioninae, Siluridae, Nemacheilidae, Oxudercidae, Acheilognathinae, and Barbinae, indicating that eDNA has a high degree of recognition for rheophilic fishes living in headwater habitats. With widespread rapids and riffles, rushing water in headwaters greatly perturbs the amount of DNA remaining in the environment, especially for water deposited in sediments. Thus, the eDNA samples collected in rapids (e.g., sites L1–L2 in the Liuxi River and Z1–Z3 in the Zeng River) contained more species‐related OTU information than the eDNA samples collected in slow‐flowing or limnetic waters of the middle and lower reaches.

An interesting finding was the difference between eDNA‐based biomarkers and biomass‐based biomarkers. The biomarkers selected by LEfSe through relative biomass preferred to screen out species with larger body sizes, such as *S. hollandi* in zone I, *X. argentea* and *P. anisitsi* in zone IV, *I. punctatus* and *A. latus* in zone VII, and *P. brachypomus* in zone VIII. This is a problem that limits the promotion and application of eDNA in a wider research area. As suggested by Rourke et al. (2022), key biotic factors influencing the effects of eDNA included the taxon examined as well as their body size, distribution, reproduction, and migration. Nevertheless, there is considerable evidence to support the use of eDNA as an ancillary tool for assessing fish population abundance and/or biomass across discrete spatiotemporal scales, following preliminary investigations to determine species‐ and context‐specific factors that influence the relationship between OTU richness and fish abundance (Bylemans et al., 2019; Doi et al., 2017). The advantages of eDNA monitoring over other approaches include reduced costs, increased efficiencies, and nonlethal sampling (Stewart, 2019).

### 4.4 Use of eDNA‐based fish monitoring to evaluate ecological status

Considering the different monitoring targets, the use of eDNA‐based fish monitoring could be summarized into four goals: (1) conducting routine investigations on the distribution and composition of fish assemblages; (2) tracing alien, protected, and endangered species that are of great indicative significance; (3) evaluating the ecological health of river systems through fish diversity; and (4) reflecting environmental changes (e.g., water pollution and habitat degradation) that may influence fish community structure. Our results demonstrated that the environmental influence on fish distribution and composition analyzed by eDNA metabarcoding was similar to that analyzed by traditional method (e.g., fish abundance and biomass obtained by electrofishing), suggesting that eDNA is a useful tool for monitoring biological communities in the field. In this study, DO, velocity, salinity, depth, COD_Mn_ (or BOD_5_), and bacterial amounts in the water were the key environmental factors influencing the fish communities. In fact, given that fishes are not only crucial components of the local food web but also high‐level predators that exhibit top‐down control effects, key environmental factors that influence fish distribution and composition may also influence food web properties (e.g., omnivory, connectivity, and stability; see Wang, Wang, Chang, et al., 2018; Wang, Wang, et al., 2019; Wang, Wang, Lin, et al., 2021). In future research, we will focus on the eDNA‐based relationships between the attributes of fish communities and those of invertebrate communities as well as on the structure and functioning of aquatic food webs.

## 5 CONCLUSIONS

We used eDNA metabarcoding to investigate the distribution and composition of fish communities in subtropical river systems. The eDNA‐based fish investigation was as effective as the electrofishing‐based investigation in both composition and diversity analyses. The relative OTU richness (%) at the taxon‐specific level could effectively distinguish the dominance of fish communities. The composition of fish communities in each spatial zone could be indicated by local biomarkers, which could help determine the specificity of the local environment in each spatial zone. The response between environmental factors and genus‐ and species‐level OTU richness was effective enough to indicate the ecological relationships between the environment and fish communities. As a novel method of assessing biodiversity, eDNA metabarcoding has great potential for use in the field monitoring of fishes and for inferring other vertebrates, invertebrates, and food webs.

## AUTHOR CONTRIBUTIONS

**Sai Wang:** Conceptualization (lead); data curation (equal); formal analysis (lead); funding acquisition (equal); investigation (equal); methodology (equal); project administration (equal); writing – original draft (lead); writing – review and editing (equal). **Dong‐Hai Wu:** Data curation (equal); formal analysis (equal); investigation (equal); methodology (equal); writing – original draft (equal). **Yong‐Duo Song:** Data curation (equal); formal analysis (equal); investigation (equal); resources (equal). **Tuan‐Tuan Wang:** Conceptualization (equal); data curation (equal); formal analysis (equal); funding acquisition (equal); methodology (equal); writing – original draft (equal); writing – review and editing (equal). **Shi‐Di Fan:** Data curation (equal); formal analysis (equal); investigation (equal); methodology (equal). **En‐Ni Wu:** Data curation (equal); investigation (equal); methodology (equal); writing – original draft (equal). **Nan‐Lin Chen:** Data curation (equal); formal analysis (equal); investigation (equal); methodology (equal). **Wen‐Tong Xia:** Methodology (equal); project administration (equal); writing – review and editing (equal). **Min N. Xu:** Funding acquisition (equal); project administration (equal); writing – review and editing (equal). **Zhong‐Bing Chen:** Conceptualization (equal); data curation (equal); formal analysis (equal). **Jing Wen:** Data curation (equal); investigation (equal); methodology (equal). **Yang Zhang:** Funding acquisition (equal); investigation (equal); project administration (equal). **Ling Mo:** Funding acquisition (equal); project administration (equal); writing – review and editing (equal). **Lei Xiang:** Funding acquisition (equal); project administration (equal).

## CONFLICT OF INTEREST STATEMENT

The authors declare that they have no competing interests.

## Supporting information


Appendix S1.

Table S2.–S6.

## Data Availability

All data supporting this study are provided as Supporting Information accompanying this manuscript. The raw sequences have been stored in the public NCBI Sequence Read Archive database under the BioProject number PRJNA1080492.
