# Supplementary material for: Application of environmental DNA metabarcoding to identify fish community characteristics in subtropical river systems
Source: Ecol Evol. 2024 May 9;14(5):e11214. doi: 10.1002/ece3.11214 (PMC11079634; doi:10.1002/ece3.11214)
Supplement: Supplementary file 1 — Appendix S1. [file ECE3-14-e11214-s001.docx]

**Supporting Information**

**Table S1** Physicochemical parameters of water quality, habitat factors, and bacterial amounts at the 38 sampling sites in the three subtropical rivers. L1 – L14, sites 1 – 14 in the Liuxi River; Z1 – Z12, sites 1 – 12 in the Zeng River; P1 – P12, sites 1 – 12 in the Pearl River.

Note: FC, Faecal coliform; MPN/L, most likely number per liter; CFU/100mL, colony forming units per 100 milliliter; TNB, total number of bacteria; DO, dissolved oxygen; EC, electrical conductivity; COD_Mn_, chemical oxygen demand determined by Mn; BOD_5_, 5-day biochemical oxygen demand; TN, total nitrogen; NH_3_−N, ammonia nitrogen; NO_3_−N, nitrate nitrogen; TP, total phosphorus; SRP, soluble reactive phosphorus; Chl-*a*, chlorophyll-*a*.

| Site | Water  depth | Flow  velocity | Discharge | Channel  width | Elevation | Riffle  area | Vegetation  cover | DO | Salinity |
| --- | --- | --- | --- | --- | --- | --- | --- | --- | --- |
|  | (m) | (m/s) | (m^3^/s) | (m) | (m) | (%) | (%) | (mg/L) | (‰) |
| L1 | 0.15 | 0.476 | 4.91 | 26 | 236.0 | 80.7 | 72.1 | 8.86 | 0.14 |
| L2 | 0.27 | 0.387 | 4.44 | 54 | 147.7 | 69.0 | 56.3 | 7.50 | 0.19 |
| L3 | 0.55 | 0.243 | 4.21 | 68 | 110.8 | 56.9 | 47.7 | 6.52 | 0.20 |
| L4 | 0.66 | 0.154 | 4.09 | 75 | 141.9 | 68.4 | 46.2 | 7.18 | 0.23 |
| L5 | 0.23 | 0.607 | 3.97 | 79 | 99.2 | 76.9 | 77.2 | 7.34 | 1.43 |
| L6 | 0.33 | 0.546 | 1.96 | 82 | 37.9 | 82.4 | 75.9 | 7.42 | 1.32 |
| L7 | 1.02 | 0.119 | 1.93 | 49 | 55.5 | 44.5 | 18.9 | 7.07 | 1.29 |
| L8 | 1.29 | 0.113 | 1.89 | 78 | 45.5 | 39.8 | 16.7 | 7.22 | 9.91 |
| L9 | 0.41 | 0.365 | 1.20 | 65 | 28.3 | 34.5 | 42.9 | 6.78 | 6.42 |
| L10 | 0.26 | 0.289 | 0.51 | 52 | 23.6 | 29.4 | 21.0 | 6.75 | 5.67 |
| L11 | 1.65 | 0.105 | 1.56 | 60 | 31.5 | 22.4 | 20.5 | 5.44 | 6.44 |
| L12 | 1.82 | 0.097 | 0.97 | 67 | 19.2 | 20.6 | 20.3 | 6.80 | 7.68 |
| L13 | 0.32 | 0.206 | 0.74 | 82 | 39.3 | 6.0 | 11.2 | 0.76 | 7.96 |
| L14 | 2.02 | 0.086 | 0.63 | 100 | 12.6 | 29.8 | 20.2 | 3.80 | 6.72 |
| Z1 | 0.14 | 0.424 | 4.38 | 32 | 149.7 | 89.4 | 72.1 | 8.16 | 0.58 |
| Z2 | 0.24 | 0.345 | 3.96 | 23 | 131.6 | 88.4 | 56.3 | 6.91 | 0.61 |
| Z3 | 0.49 | 0.217 | 3.75 | 19 | 98.7 | 77.6 | 47.7 | 6.01 | 0.39 |
| Z4 | 0.58 | 0.137 | 3.64 | 15 | 126.4 | 69.8 | 46.2 | 6.62 | 1.43 |
| Z5 | 0.21 | 0.359 | 3.54 | 20 | 88.4 | 38.2 | 57.2 | 6.76 | 2.14 |
| Z6 | 0.29 | 0.323 | 1.74 | 26 | 33.8 | 40.5 | 33.2 | 6.84 | 1.42 |
| Z7 | 0.91 | 0.106 | 1.72 | 59 | 49.5 | 30.1 | 30.1 | 6.51 | 3.64 |
| Z8 | 1.15 | 0.101 | 1.69 | 20 | 40.5 | 42.5 | 28.7 | 6.65 | 6.97 |
| Z9 | 0.36 | 0.325 | 1.07 | 11 | 25.2 | 33.1 | 27.1 | 6.25 | 9.77 |
| Z10 | 0.23 | 0.257 | 0.45 | 37 | 7.0 | 28.5 | 25.4 | 6.22 | 10.53 |
| Z11 | 1.47 | 0.094 | 1.39 | 62 | 6.0 | 32.5 | 23.8 | 5.01 | 11.28 |
| Z12 | 1.62 | 0.086 | 0.86 | 41 | 5.0 | 23.1 | 20.3 | 6.27 | 11.94 |
| P1 | 1.76 | 0.029 | 0.57 | 110 | 11.0 | 18.4 | 20.3 | 3.50 | 8.90 |
| P2 | 1.63 | 0.030 | 0.54 | 115 | 10.9 | 17.4 | 20.3 | 2.83 | 8.94 |
| P3 | 1.50 | 0.032 | 0.51 | 118 | 8.8 | 18.9 | 20.3 | 3.24 | 10.51 |
| P4 | 1.04 | 0.047 | 1.10 | 120 | 7.6 | 15.4 | 21.1 | 3.32 | 10.55 |
| P5 | 1.97 | 0.128 | 6.00 | 181 | 3.4 | 16.4 | 10.3 | 4.06 | 12.76 |
| P6 | 2.21 | 0.101 | 2.96 | 118 | 2.4 | 3.3 | 8.5 | 4.20 | 12.54 |
| P7 | 2.47 | 0.082 | 2.91 | 98 | 2.0 | 2.8 | 6.2 | 2.99 | 12.44 |
| P8 | 2.77 | 0.076 | 2.86 | 79 | 1.7 | 2.3 | 6.0 | 4.08 | 14.61 |
| P9 | 3.10 | 0.162 | 1.82 | 90 | 1.1 | 2.3 | 5.6 | 3.82 | 15.74 |
| P10 | 3.47 | 0.068 | 0.77 | 101 | 1.0 | 4.9 | 5.2 | 3.91 | 15.40 |
| P11 | 3.89 | 0.023 | 2.36 | 124 | 0.8 | 1.4 | 5.6 | 4.68 | 18.90 |
| P12 | 4.36 | 0.024 | 1.46 | 305 | 0.3 | 0.8 | 0.3 | 5.66 | 17.90 |
| Continued- | | | | | | | | | |
| Site | EC | COD_Mn_ | BOD_5_ | Chl-*a* | TN | NH_3_-N | NO_3_-N | TP | SRP |
|  | (μS/cm) | (mg/L) | (mg/L) | (mg/L) | (mg/L) | (mg/L) | (mg/L) | (mg/L) | (mg/L) |
| L1 | 28.7 | 0.14 | 1.11 | 0.74 | 1.89 | 0.59 | 1.22 | 0.012 | 0.005 |
| L2 | 55.7 | 0.18 | 1.28 | 0.66 | 1.83 | 0.57 | 1.18 | 0.016 | 0.007 |
| L3 | 43.5 | 0.86 | 2.27 | 1.32 | 1.84 | 0.57 | 1.19 | 0.117 | 0.051 |
| L4 | 20.4 | 1.05 | 1.51 | 1.87 | 1.64 | 0.51 | 1.06 | 0.037 | 0.016 |
| L5 | 130.2 | 0.31 | 1.45 | 1.17 | 1.20 | 0.37 | 0.78 | 0.103 | 0.045 |
| L6 | 56.5 | 0.40 | 1.64 | 0.94 | 1.89 | 0.59 | 1.22 | 0.081 | 0.035 |
| L7 | 65.0 | 2.85 | 2.10 | 3.34 | 1.27 | 0.40 | 0.82 | 0.223 | 0.097 |
| L8 | 37.7 | 6.89 | 2.24 | 7.40 | 4.17 | 1.30 | 2.70 | 0.135 | 0.059 |
| L9 | 130.4 | 0.51 | 3.25 | 1.57 | 4.23 | 1.32 | 2.74 | 0.268 | 0.117 |
| L10 | 130.0 | 0.88 | 3.47 | 1.04 | 4.28 | 1.34 | 2.77 | 0.295 | 0.128 |
| L11 | 115.8 | 7.58 | 1.73 | 4.53 | 4.22 | 1.32 | 2.73 | 0.116 | 0.050 |
| L12 | 38.9 | 12.20 | 2.69 | 9.02 | 4.81 | 1.50 | 3.11 | 0.187 | 0.081 |
| L13 | 354.2 | 21.20 | 5.15 | 1.02 | 9.45 | 2.95 | 6.12 | 1.365 | 0.594 |
| L14 | 287.5 | 5.12 | 4.91 | 2.93 | 7.05 | 2.20 | 4.56 | 0.806 | 0.351 |
| Z1 | 40.5 | 2.90 | 2.65 | 0.72 | 1.66 | 0.52 | 1.07 | 0.281 | 0.088 |
| Z2 | 54.3 | 5.40 | 2.63 | 0.64 | 2.92 | 0.91 | 1.89 | 0.231 | 0.089 |
| Z3 | 76.5 | 4.11 | 2.61 | 1.28 | 3.56 | 1.11 | 2.30 | 0.205 | 0.089 |
| Z4 | 109.8 | 3.46 | 2.60 | 1.82 | 4.19 | 1.31 | 2.71 | 0.180 | 0.090 |
| Z5 | 85.4 | 2.81 | 3.40 | 1.13 | 4.04 | 1.26 | 2.62 | 0.240 | 0.150 |
| Z6 | 116.3 | 2.76 | 2.70 | 0.91 | 4.83 | 1.51 | 3.13 | 0.300 | 0.200 |
| Z7 | 165.4 | 2.84 | 3.00 | 3.24 | 3.22 | 1.00 | 2.08 | 0.170 | 0.080 |
| Z8 | 231.2 | 2.91 | 2.30 | 7.19 | 4.38 | 1.37 | 2.84 | 0.170 | 0.100 |
| Z9 | 143.2 | 3.32 | 2.55 | 1.53 | 3.17 | 0.99 | 2.05 | 0.175 | 0.065 |
| Z10 | 276.8 | 2.48 | 2.80 | 1.01 | 1.96 | 0.61 | 1.27 | 0.180 | 0.030 |
| Z11 | 328.1 | 2.87 | 2.75 | 4.39 | 2.94 | 0.92 | 1.90 | 0.175 | 0.025 |
| Z12 | 449.0 | 3.40 | 2.70 | 8.75 | 3.92 | 1.22 | 2.54 | 0.170 | 0.020 |
| P1 | 297.4 | 37.10 | 3.86 | 2.68 | 5.84 | 1.82 | 3.78 | 0.330 | 0.215 |
| P2 | 307.7 | 29.60 | 3.33 | 4.66 | 5.24 | 1.64 | 3.39 | 0.410 | 0.148 |
| P3 | 318.3 | 24.50 | 2.80 | 6.09 | 4.64 | 1.45 | 3.00 | 0.980 | 0.080 |
| P4 | 329.3 | 25.30 | 2.70 | 8.92 | 4.42 | 1.38 | 2.86 | 0.870 | 0.085 |
| P5 | 544.2 | 24.60 | 2.19 | 6.57 | 2.46 | 0.77 | 1.59 | 0.310 | 0.196 |
| P6 | 690.8 | 22.20 | 2.48 | 7.66 | 5.88 | 1.83 | 3.81 | 0.300 | 0.220 |
| P7 | 725.2 | 19.00 | 3.18 | 7.12 | 5.02 | 1.56 | 3.25 | 0.285 | 0.175 |
| P8 | 844.4 | 11.20 | 3.38 | 7.39 | 4.58 | 1.43 | 2.97 | 0.278 | 0.153 |
| P9 | 954.1 | 10.70 | 4.91 | 7.25 | 4.37 | 1.36 | 2.83 | 0.274 | 0.141 |
| P10 | 1064.1 | 13.10 | 5.24 | 7.32 | 4.15 | 1.29 | 2.69 | 0.270 | 0.130 |
| P11 | 1254.2 | 12.50 | 2.61 | 7.29 | 6.11 | 1.91 | 3.96 | 0.220 | 0.120 |
| P12 | 1766.0 | 11.60 | 4.06 | 7.31 | 5.14 | 1.60 | 3.33 | 0.178 | 0.089 |
| Continued- | | | | | | | | | |
| Site | FC | Coliform | TNB | Cu | Zn | Se | As | Hg | Cd |
|  | (MPN/L) | (MPN/L) | (CFU/100mL) | (μg/L) | (μg/L) | (μg/L) | (μg/L) | (μg/L) | (μg/L) |
| L1 | 2600 | 30000 | 5800 | 13.3 | 53.3 | 0.22 | 12.2 | 0.08 | 0.21 |
| L2 | 5400 | 36500 | 5700 | 35.7 | 57.6 | 0.35 | 13.6 | 0.15 | 0.58 |
| L3 | 6800 | 39750 | 5650 | 36.2 | 55.2 | 0.42 | 14.2 | 0.19 | 0.77 |
| L4 | 7500 | 41375 | 5625 | 36.7 | 54.3 | 0.45 | 14.6 | 0.21 | 0.86 |
| L5 | 7850 | 42188 | 5600 | 38.1 | 53.3 | 0.48 | 14.9 | 0.22 | 0.95 |
| L6 | 8200 | 43000 | 7000 | 50.5 | 51.0 | 0.57 | 16.5 | 0.29 | 1.04 |
| L7 | 4850 | 39500 | 5600 | 56.7 | 48.6 | 0.44 | 13.9 | 0.32 | 0.73 |
| L8 | 7800 | 81000 | 6350 | 62.9 | 122.4 | 0.32 | 11.2 | 0.35 | 0.43 |
| L9 | 6500 | 78000 | 7100 | 67.6 | 157.1 | 0.31 | 10.3 | 0.27 | 0.39 |
| L10 | 5200 | 76500 | 5100 | 51.9 | 174.3 | 0.30 | 9.5 | 0.20 | 0.34 |
| L11 | 5950 | 75750 | 6700 | 94.8 | 191.4 | 0.62 | 17.3 | 0.32 | 1.10 |
| L12 | 6700 | 75000 | 8400 | 47.1 | 210.5 | 0.39 | 11.9 | 0.32 | 0.51 |
| L13 | 8200 | 86000 | 12684 | 65.2 | 159.0 | 0.49 | 14.6 | 0.31 | 0.83 |
| L14 | 10000 | 72500 | 6650 | 74.3 | 107.1 | 0.54 | 16.0 | 0.31 | 1.00 |
| Z1 | 3175 | 37750 | 7900 | 11.4 | 185.7 | 0.48 | 14.3 | 0.22 | 0.40 |
| Z2 | 2338 | 36875 | 4500 | 54.3 | 207.6 | 0.46 | 13.8 | 0.21 | 0.27 |
| Z3 | 1919 | 36438 | 6200 | 49.5 | 218.1 | 0.45 | 13.5 | 0.20 | 0.20 |
| Z4 | 1500 | 36000 | 8550 | 47.1 | 223.8 | 0.44 | 13.3 | 0.19 | 0.14 |
| Z5 | 2000 | 29000 | 8875 | 44.8 | 229.0 | 0.71 | 20.3 | 0.32 | 1.10 |
| Z6 | 2600 | 33000 | 9038 | 61.4 | 192.4 | 0.69 | 17.5 | 0.35 | 1.21 |
| Z7 | 5900 | 22000 | 9200 | 104.8 | 173.8 | 0.24 | 15.8 | 0.26 | 0.17 |
| Z8 | 2000 | 35000 | 9700 | 15.7 | 164.3 | 0.27 | 11.3 | 0.15 | 0.23 |
| Z9 | 1100 | 30500 | 7100 | 15.2 | 160.0 | 0.25 | 12.4 | 0.11 | 0.23 |
| Z10 | 3650 | 26000 | 8200 | 14.8 | 155.2 | 0.22 | 13.4 | 0.07 | 0.23 |
| Z11 | 6200 | 68000 | 9300 | 13.8 | 246.2 | 0.23 | 11.8 | 0.12 | 0.21 |
| Z12 | 4100 | 110000 | 8500 | 12.9 | 256.7 | 0.24 | 10.2 | 0.17 | 0.20 |
| P1 | 11000 | 59000 | 6625 | 79.0 | 101.4 | 0.56 | 16.6 | 0.30 | 1.08 |
| P2 | 11500 | 67000 | 6613 | 81.0 | 95.2 | 0.57 | 17.0 | 0.30 | 1.12 |
| P3 | 11750 | 75000 | 6606 | 83.3 | 233.3 | 0.59 | 17.3 | 0.30 | 1.16 |
| P4 | 12000 | 94000 | 6600 | 64.3 | 142.4 | 0.51 | 15.3 | 0.25 | 0.65 |
| P5 | 18120 | 113250 | 7700 | 95.0 | 289.0 | 0.72 | 22.5 | 0.34 | 1.43 |
| P6 | 11778 | 129860 | 8456 | 102.1 | 317.9 | 0.86 | 24.9 | 0.44 | 1.57 |
| P7 | 9815 | 109475 | 10570 | 78.4 | 240.1 | 0.66 | 21.0 | 0.48 | 1.10 |
| P8 | 7852 | 89090 | 8456 | 53.9 | 161.7 | 0.48 | 16.9 | 0.52 | 0.65 |
| P9 | 8985 | 101170 | 9589 | 54.7 | 153.1 | 0.47 | 15.6 | 0.41 | 0.59 |
| P10 | 10117 | 113250 | 10117 | 55.4 | 143.8 | 0.45 | 14.3 | 0.30 | 0.51 |
| P11 | 12382 | 141940 | 10721 | 143.1 | 352.3 | 0.94 | 26.1 | 0.48 | 1.66 |
| P12 | 3926 | 45300 | 7701 | 71.1 | 215.0 | 0.59 | 18.0 | 0.49 | 0.77 |

**Figure S1** Rarefaction curves based on the number of reads on the x-axis against the number of OTUs on the y-axis. (A) Rarefaction curves for 14 sampling sites in the Liuxi River. (B) Rarefaction curves for 12 sampling sites in the Zeng River. (C) Rarefaction curves for 12 sampling sites in the Pearl River.

**Figure S2** Heatmap of the correlations between environmental factors and the eDNA-based fish community composition. Correlation coefficients are illustrated using progressive colours from blue (lower values) to red (higher values). Cluster analysis of the fish species and environmental factors based on the correlation matrix are listed on the left and top of the graph, respectively. (A) Correlation heatmap based on environmental factors and relative percentage (%) of OTU reads at the order level. (B) Correlation heatmap based on environmental factors and relative percentage (%) of OTU reads at the family level. (C) Correlation heatmap based on environmental factors and relative percentage (%) of OTU reads at the genus level. Water depth (m); Channel width (m); Flow velocity (m/s); Discharge (m^3^/s); Elevation (m), Riffle area (%); Vegetation cover (%); FC, faecal coliform (MPN/L, most likely number per liter); TNB, total number of bacteria (CFU/100 mL, colony forming units per 100 milliliter); DO, dissolved oxygen (mg/L); EC, electrical conductivity (μS/cm); COD_Mn_, chemical oxygen demand determined by Mn (mg/L); BOD_5_, 5-day biochemical oxygen demand (mg/L); TN, total nitrogen (mg/L); NH_3_−N, ammonia nitrogen (mg/L); NO_3_−N, nitrate nitrogen (mg/L); TP, total phosphorus (mg/L); SRP, soluble reactive phosphorus (mg/L); Chl-*a*, chlorophyll-*a* (mg/L); Salinity (‰); and Heavy metal (μg/L), including Cu, Zn, Se, As, Hg, and Cd. "*", "**", and "***" indicate significant differences at *p* < 0.05, *p* < 0.01, and *p* < 0.001, respectively.
